# The risk for depression in patients with ankylosing spondylitis: a population-based cohort study

**DOI:** 10.1186/s13075-014-0418-z

**Published:** 2014-08-18

**Authors:** Jorit JL Meesters, Ann Bremander, Stefan Bergman, Ingemar F Petersson, Aleksandra Turkiewicz, Martin Englund

**Affiliations:** Epidemiology and Register Centre South Skåne, Skåne University Hospital, Lund, Sweden; Department of Orthopaedics, Leiden University Medical Centre, Leiden, the Netherlands; Department of Clinical Sciences, Lund, Section of Rheumatology, Lund University, Lund, Sweden; Spenshult, Research and Development Center, Oskarström, Sweden; School of Business and Engineering, Department of Exercise Physiology, Biomechanics and Health, Halmstad University, Halmstad, Sweden; Orthopaedics, Clinical Sciences Lund, Lund University, Lund, Sweden; Clinical Epidemiology Research & Training Unit, Boston University School of Medicine, Boston, MA USA

## Abstract

**Introduction:**

Depression is frequent in ankylosing spondylitis (AS) patients. However, epidemiological data about the potential increase in risk are lacking. This study compares the rate of doctor-diagnosed depression in a well defined cohort of AS patients to the general population seeking care.

**Methods:**

The Skåne Healthcare Register comprises healthcare data of each resident in Region Skåne, Sweden (population 1.2 million), including ICD-10 diagnoses. Using physician coded consultation data from years 1999 to 2011, we calculated depression consultation rates for all AS patients. We obtained standardized depression-rate ratios by dividing the observed depression rate in AS patients by the expected rate based on the corresponding age- and sex-specific rates of depression in the general population seeking care. A ratio >1 equals a higher rate of depression among AS patients.

**Results:**

The AS cohort consisted of 1738 subjects (65% men) with a mean age of 54 years. The reference population consisted of 967,012 subjects. During the 13-year observation period 10% (n = 172) of the AS cohort had a doctor-diagnosed depression compared to 6% (n = 105) to be expected. The standardized estimate of depression-rate ratio was 1.81 (95% confidence interval 1.44 to 2.24) in women men and 1.49 (1.20 to 1.89) in men.

**Conclusions:**

The rate of doctor-diagnosed depression is increased about 80% in female and 50% in male AS patients. Future challenges are to timely identify and treat the AS patients who suffer from depression.

## Introduction

The prevalence of ankylosing spondylitis (AS) in the Western world is estimated to be 0.1 to 0.2%, with an estimated incidence of 7/100,000 per year [1–3]. Due to the nature of the disease, AS typically affects physical function, quality of life [4,5], and work productivity [6,7]. Comorbid conditions, including uveitis, inflammatory bowel and cardiovascular disease, contribute to the prognosis and total burden of AS [8].

Clinical depression has also been reported to be more frequent in chronic diseases [9], rheumatic diseases in general [10] and AS [11–13]. However, an estimate of the actual increase in risk, and its age- and/or gender-dependency, of depression in AS patients is lacking. Therefore, our objective was to study consultation rates of doctor-diagnosed depression in female and male AS patients compared to the general population seeking healthcare in a population-based open cohort study. We hypothesized that the consultation rates of depression are significantly higher in AS patients.

## Methods

### The Skåne healthcare register (SHR)

Detailed information about the SHR is provided in previous articles [8,14]. In brief, each single healthcare consultation in this region generates data entries by the healthcare provider that are transferred to central databases by the patient’s unique 10-digit personal identification number. Data includes information about date of consultation and international classification of disease (ICD)-10 diagnostic codes by the responsible physician, drawn from the patients’ computer-based medical records.

### The Swedish Population Register

The Swedish Population Register is the civil registration of vital events (for example, births, deaths, removal) of Swedish inhabitants. For this study, we linked SHR data via the personal identification number to add information on date of death and residency.

### Inclusion criteria for the AS cohort

We required all AS cases to be at least 20 years of age and to be resident in Skåne Region by the first healthcare visit in the SHR between 1999 and 2011. Further, our case definition required a clinic visit with a diagnosis of AS (ICD-10 code M45) made by a physician during the years 1999 to 2011 (13 calendar years), at least once at a rheumatology clinic or internal medicine clinic, or at least twice at any other clinic including general practice. The date of fulfilling the above definition marked the beginning of the exposure time. A previous validation of spondyloarthritis at large, showed 98% of patients identified via the register to be valid spondyloarthritis patients [2].

### Inclusion criteria for the reference population

In this article the general population seeking healthcare is regarded as the reference population. For the AS cohort, we required all subjects in the reference population to be residents in Skåne Region and aged 20 years or older by the first healthcare visit registered in the SHR between 1999 and 2011. Then we required all of the subjects to have had at least one clinic visit between 1 January 1999 and 31 December 2011, with any diagnosis by any physician in the SHR, to minimize confounding.

### Outcome

For the reference population we calculated each single subject’s person-time from the first healthcare visit until one of the following four events had occurred for the first time within the observation period from the first visit until 31 December 2011, in the following order of priority: 1) occurrence of doctor-diagnosed depressive episode (ICD-10 code F32) or recurrent depressive disorder (F33); 2) death; 3) end of the observation period if the subject was still a resident in the county by that time; or 4) date of relocation outside of the region. The same procedure was applied to the AS patients, where person-time was calculated from the date of fulfilling the AS definition.

### Statistical analysis

#### Observed depression rate for AS patients

We calculated observed consultation rates for doctor-diagnosed depression (depression rates) among patients fulfilling our AS case criteria. The total number of AS patients having had the diagnosis within the study period formed the numerator of the rate, and the AS patients’ added person-time formed the denominator of the rate. The age of all subjects was defined as the age at the mean follow-up time. We calculated rates for each age and sex stratum (using 5-year age categories).

#### Expected depression rate for AS patients

We calculated the expected doctor-diagnosed depression rate as if the rate in the general population seeking healthcare prevailed, using age and sex of the AS patients as the standard. Hence, using data from the SHR, we calculated the sum of each resident’s person-time and counted all subjects diagnosed with the first occurrence of depression during the study period. We then calculated the expected depression rate by multiplying the stratum-specific rates in the reference population with the appropriate age and sex weightings from the AS cohort. Then we added the stratum-specific products to obtain the age- and sex-standardized rate.

#### Standardized depression-rate ratio and absolute rate difference

We calculated age- and sex-standardized depression-rate ratio by dividing the observed morbidity rate in the AS cohort by their expected morbidity rate. A rate ratio >1 equals a higher depression rate in the AS cohort than in the general population seeking healthcare. We calculated 95% CI for the rate ratios using the method described by Breslow [15]. We also calculated the absolute depression-rate difference (observed rate minus expected rate) to provide results of excess doctor-diagnosed depression on the absolute scale as well.

#### Gender differences

To evaluate the difference in depression-rate ratio in women and men for AS patients and the reference population, respectively, we also calculated the ratio of these rate ratios with their 95% CI.

### Diabetes mellitus

To facilitate interpretation of the results obtained for the AS patients, we also evaluated patients with another chronic disease reported to be associated with increased risk of depression, insulin-dependent and non-insulin-dependent diabetes mellitus (ICD-10 code E10 or E11) [16]. Our case definition for diabetes required a clinic visit, with a physician's diagnosis of diabetes at least once at an endocrinology or internal medicine clinic, or at least twice at any other clinic, including general practice.

### Ethics statement

The study was conducted according to the Declaration of Helsinki and was approved by the Ethical Review Board of Lund University, Sweden. The population in the Skåne region was informed of the study via regional news press and offered the opportunity to opt out, a process sanctioned by the Ethical Review Board. Thus, no individually signed informed consent forms were required according to the decision from the Ethical Review Board (a common procedure in Sweden for population-based register studies).

## Results

The number of residents (aged ≥20 years) in Skåne Region who fulfilled our AS case criteria during our 13-year study period was 1,738 (64.4% men), mean age (SD) 54.5 (14.3) years (1,275 AS patients (73%) had been diagnosed at a rheumatology or internal medicine clinic). The total adult general population seeking healthcare during the same period consisted of 967,012 subjects (47.6% men).

During the study period 10% (n = 172) of the AS cohort had at least one episode of doctor-diagnosed depression compared to the expected 6% (n = 105) based on age- and sex-standardized data from the reference population. The standardized depression-rate ratio in subjects with AS (Table 1) was significantly elevated (1.63; 95% CI 1.40, 1.89). The standardized depression-rate ratio in women (1.81; 95% CI 1.44, 2.24) and in men (1.49; 95% CI 1.20, 1.89) were both elevated. However, the age-standardized ratio between these rate ratios (women versus men) was not significantly different (1.20; 95% CI 0.89, 1.62), implying no substantial interaction between AS and sex for the risk of doctor-diagnosed depression.

**Table 1:** **Doctor-diagnosed depression in ankylosing spondylitis (AS) patients (n = 1,738) compared with the general population seeking healthcare (n = 967,012)**

	**Observed**			**Expected**				
	**Person time (years)**	**Number of cases** ^**1**^	**Depression rate** ^**2**^	**Number of cases**	**Depression rate** ^**2**^	**Absolute rate differences** ^**2**^	**Rate ratio** ^**3**^	**95% CI**
Men, all	6,983	89	1,275	60	853	421	**1.49**	1.20, 1.89
≤50 years	2,879	42	1,376	27	933	443	**1.47**	1.10, 1.93
>50 years	4,104	47	1,155	31	759	396	**1.52**	1.07, 2.10
Women, all	3,259	83	2,547	46	1,409	1,138	**1.81**	1.44, 2.24
≤50 years	1,274	39	2,825	20	1,546	1,279	**1.83**	1.35, 2.42
>50 years	1,984	44	2,244	25	1,260	984	**1.78**	1.24, 2.48
Total, all	10,242	172	1,679	105	1,030	649	**1.63**	1.40, 1.89
≤50 years	4,153	83	1,825	47	1,123	702	**1.63**	1.32, 1.98
>50 years	6,089	89	1,512	56	923	588	**1.64**	1.28, 2.06

^1^AS patients diagnosed with depression (ICD-10 code F32 or F33) in 1999 to 2011. ^2^Per 100,000 person-years. ^3^Bold figures indicate statistically significant results.

The absolute rate difference (observed minus expected) for doctor-diagnosed depression in subjects with AS was 649 per 100,000 person-years. The absolute rate difference was greater in women (1,138 per 100,000 person-years) compared to men (421 per 100,000 person-years). However, this difference is explained by the much higher observed depression rate in women in general compared to men (Table 1). The rate of doctor-diagnosed depression in women in the reference population was highly elevated compared to men (standardized depression rate ratio = 1.66; 95% CI 1.63, 1.68), that is, women had a 66% higher increased rate than men. The standardized depression-rate ratio in subjects with diabetes mellitus was significantly elevated (1.67; 95% CI 1.63, 1.71).

## Discussion

In a cohort study design, using comprehensive population-based epidemiologic data based on physicians’ diagnostic coding, our hypothesis that consultation rates of depression are significantly higher in AS patients was confirmed. We found an 80% increased rate of doctor-diagnosed depression in women with AS and 50% in men with AS compared to the general population seeking care. The higher rate in women is due to general sex differences and thus is not so much AS-specific. Although these findings come as no surprise, population-based epidemiologic estimates of the actual increase in risk and potential age and gender aspects are lacking. These findings enhance our understanding of the total burden for AS patients and may contribute to improved health assessment and treatment planning [17].

Prior studies have suggested a relationship between depression and rheumatic diseases [10] and AS [11,13]. In the present study we found evidence that the risk for depression is higher in AS patients, and the increase is in line with patients with diabetes mellitus. We further found the absolute rate of doctor-diagnosed depression to be much higher in women than men, both in the general population and in AS patients. These findings are in line with literature regarding prevalence of depression in the general population [18] and in patients with rheumatic diseases [10].

In our study, we did not include person-time for residents not seeking healthcare in the study time frame. Including such person-time would elevate the rate ratios even further. This decision was made to compensate for bias caused by tendency to consult (irrespective of reason) being higher in patients with previous consultations. Still, we cannot fully exclude the possibility of residual doctors’ work-up bias towards patients with a chronic disease such as AS. However, we find this unlikely because depression is not a typical diagnosis for which patients are screened, unlike hypertension, hypercholesterolemia, or diabetes. Furthermore, we included both prevalent and incident disease, both for the AS diagnosis and for depression, and data have not been controlled for possible confounding factors other than age and sex, and propensity to seek care. Further, in this study we could not relate the risk to disease duration, disease severity or other covariates, therefore residual confounding is possible. Moreover, the use of diagnostic codes based on physicians coding in everyday practice might pose a threat to the validity. However, the accuracy of the diagnostic coding from the SHR has previously been validated for spondyloarthritis, as well as specifically for psoriatic arthritis and psoriasis, with positive predictive values consistently exceeding 81% [19]. The diagnostic coding of depression could not be externally verified, for example, by review of medical records, due to restrictions in the approval of our institutional review board.

## Conclusions

Based on our findings, we conclude that the consultation rate for depression is increased by more than 60% in AS patients compared to the background population seeking care, with women having somewhat larger increase in risk. One future challenge is to timely identify AS patients with depression. Research should focus on finding out which patient-reported outcome measures are most suitable to identify a depressive disorder in AS patients. The Hospital Anxiety and Depression Scale, the Hamilton Rating Scale for Depression, the Beck Depression Inventory, or the Center for Epidemiologic Studies Depression Scale [10] could be considered, as these have previously been used to asses depression in studies of rheumatic disorders.

## Abbreviations

AS: ankylosing spondylitis
ICD: International classification of diseases
SHR: Skåne healthcare register
